# X-ray Photoelectron Spectroscopy Analysis of Nafion-Containing Samples: Pitfalls, Protocols, and Perceptions of Physicochemical Properties

**DOI:** 10.1021/acs.jpcc.4c00872

**Published:** 2024-05-10

**Authors:** Michael J. Dzara, Kateryna Artyushkova, Jayson Foster, Hamideh Eskandari, Yechuan Chen, Scott A. Mauger, Plamen Atanassov, Kunal Karan, Svitlana Pylypenko

**Affiliations:** † Department of Chemistry, Colorado School of Mines, Golden, Colorado 80401, United States; ‡ Physical Electronics Inc., Chanhassen, Minnesota 55317, United States; § Department of Chemical and Petroleum Engineering, University of Calgary, Calgary, Alberta T2N 1N4, Canada; ∥ Department of Chemical & Biomolecular Engineering, University of California Irvine, Irvine, California 92697, United States; ⊥ National Renewable Energy Laboratory, Materials Science Center, Golden, Colorado 80401, United States

## Abstract

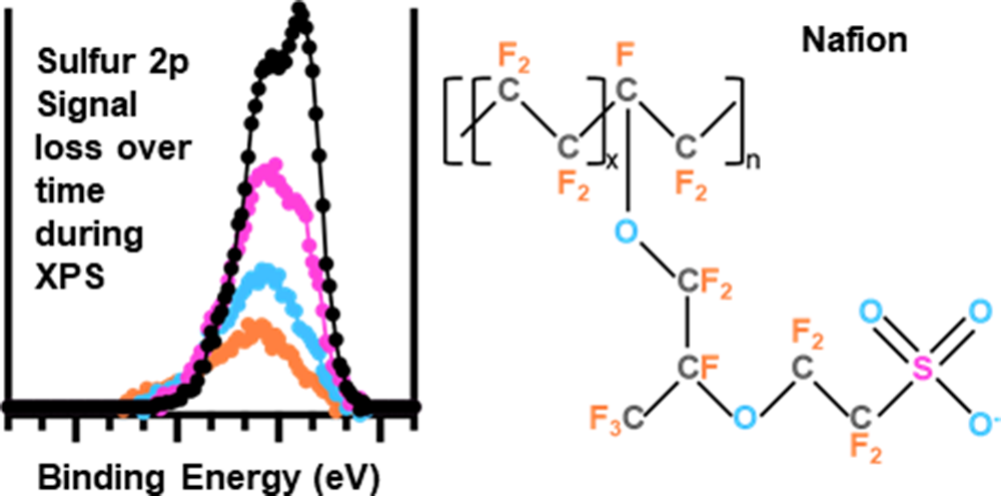

X-ray photoelectron spectroscopy (XPS) is one of the most common techniques used to analyze the surface composition of catalysts and support materials used in polymer electrolyte membrane (PEM) fuel cells and electrolyzers, providing important insights for further improvement of their properties. Characterization of catalyst layers (CLs) is more challenging, which can be at least partially attributed to the instability of ionomer materials such as Nafion during measurements. This work explores the stability of Nafion during XPS measurements, illuminating and addressing Nafion degradation concerns. The extent of Nafion damage as a function of XPS instrumentation, measurement conditions, and sample properties was evaluated across multiple instruments. Results revealed that significant Nafion damage to the ion-conducting sulfonic acid species (>50% loss in sulfur signal) may occur in a relatively short time frame (tens of minutes) depending on the exact nature of the sample and XPS instrument. This motivated the development and validation of a multipoint XPS data acquisition protocol that minimizes Nafion damage, resulting in reliable data acquisition by avoiding significant artifacts from Nafion instability. The developed protocol was then used to analyze both thin film ionomer samples and Pt/C-based CLs. Comparison of PEM fuel cell CLs to Nafion thin films revealed several changes in Nafion spectral features attributed to charge transfer due to interaction with conductive catalyst and support species. This study provides a method to reliably characterize ionomer-containing samples, facilitating fundamental studies of the catalyst-ionomer interface and more applied investigations of structure-processing-performance correlations in PEM fuel cell and electrolyzer CLs.

## 1 Introduction

Polymer electrolyte membrane (PEM) fuel cells (FCs) and water electrolyzers (WEs) are very active research areas due to their current commercial relevance and prospects as energy conversion technologies within a sustainable energy economy. While these technologies have been sufficiently developed to reach a commercial breakthrough in transportation applications and in the energy conversion sector at the megawatt scale, there are still a myriad of opportunities for improvements in PEM device performance and/or reductions in cost. The design and fabrication of catalyst layers (CLs) is one such area where further research is needed to drive device improvements. CLs typically consist of a catalyst (most often a platinum-group metal nanoparticle), an ionomer (ion-conducting polymer, most often Nafion, the commercial name for a family of perfluorinated sulfonic acid polymers produced by Chemours), and in many cases, an electron-conducting support (most often a carbonaceous material).^1−3^ It is crucial to understand the underlying material interactions between the ionomer and the catalyst (and when relevant, the ionomer and support) and how they relate to the fundamental properties that determine PEM device performance such as ion and mass transport, catalyst utilization, and degradation rates.^1,4−9^ Nafion has been more thoroughly characterized at the bulk scale (on the order of tens-hundreds of microns in thickness) due to its common use as the PEM in fuel cells and electrolyzers.^10−12^ However, Nafion ionomer exists as much thinner (1–100 nanometers) films coating the catalyst/support within a CL, and a number of studies have shown that bulk, surface, and interfacial properties of Nafion thin films are thickness-dependent, indicating structural change at the nanoscale.^13,14^ Additional comprehensive materials characterization studies focused on examining the interactions between the catalyst-ionomer and support-ionomer as a function of processing, fabrication, and testing are needed to drive further advancements in PEMFC and PEMWE performance.

A theme presents itself throughout the literature featuring the characterization of Nafion in PEM CL sand thin films. Nafion is a very dynamic material, and its responsiveness to external stimuli clouds the ability to definitively investigate catalyst-ionomer interactions and confidently correlate characterization results to CL properties and performance. Electron microscopy has been employed frequently to uncover morphological properties and constituent distribution within the CL of interest.^15−18^ Transmission electron microscopy revealed that Nafion forms thin (<5 nm) coatings on platinum nanoparticles on a carbon support (Pt/C),^19^ and demonstrated that distribution and thickness of ionomer in catalyst layers vary depending on ink composition,^20,21^ processing methods, and catalyst and support properties.^22−25^ Limitations of electron microscopy methods include challenging sample preparation and the possibility of Nafion dynamical properties when irradiated by an electron beam. Nevertheless, such studies are extremely valuable as a feedback loop toward the design of peak performance PEM electrodes and in processing and fabrication efforts. These findings also sparked fundamental research into model Nafion thin films of different thicknesses.^26^ X-ray techniques including computed tomography and scattering measurements have been used to investigate both catalyst layers and thin films, identifying the size and shape of Nafion agglomerations, and more.^18,27−29^ An example is provided with small-angle X-ray scattering, as ex situ and humidified in situ measurements have provided contextual information on ionomer interfaces as a function of hydrophilicity of the support material.^30^ Successful studies of CL and Nafion thin film surfaces have also been conducted with atomic force microscopy and other scanning probe techniques, yielding valuable knowledge of surface morphology and topology of samples; these studies are particularly insightful when conducted under relevant conditions such as humidification.^31−33^

X-ray photoelectron spectroscopy (XPS) is a particularly attractive technique for the study of CLs due to its sensitivity to the surface chemical state, availability of instruments with in situ capabilities, and the relative ease of sample preparation compared to other techniques. XPS has been used to investigate Nafion surface chemical state in membranes, and less frequently, in thin films and as part of a CL.^15,34−39^ While the above literature paints a promising picture of the field’s efficacy in understanding ionomer properties and interactions within PEM electrodes, there are also a number of reports that tell a much more cautionary tale, particularly when using X-ray sources; Nafion degrades under X-ray exposure in some cases, which may significantly impact studies focused on catalyst-ionomer interfaces.^40−43^ A report on in operando imaging of a fuel cell concluded that upon exposure to synchrotron X-ray radiation, the performance of their PEM fuel cell degraded irreversibly within minutes, calling into question the compatibility of synchrotron X-rays and PEM materials.^41^ Indeed, early attempts to characterize Nafion with XPS showed that some Nafion samples were not stable throughout the measurement with changes in spectra occurring as a function of measurement time. However, the literature on characterization of Nafion with XPS has been historically inconclusive on the degree of damage imposed by X-ray exposure likely due to variability in instrumentation, length and strength of X-ray exposure, Nafion film thickness, or substrate composition.^40,42−44^ For example, Schulz et al. reported in 1999 significant changes in the C 1s spectrum for a H_2_O_2_-cleaned 175 μm Nafion film (Nafion 117, Dupont) compared to a different Nafion film with ion-etching over the course of 20 h at 300 W. The authors suspected changes in XPS spectral intensity was primarily due to the length of X-ray exposure. In 2005, a Surface Science Spectra article reported a loss of sulfonic acid signal in both the S 2p and O 1s regions for a 50 μm thick film exposed to 200 and 100 W X-ray source and a 3 eV electron flood gun used for charge neutralization. Additionally, the authors include a comment that the effects of additional surface charging were observed as X-ray exposure increased.^42^ However, in 2007 when Chen et al. were investigating membrane degradation under X-ray radiation, they observed a lack of discernible change to the C 1s, O 1s, F 1s, and S 2p core levels after 2 h of X-ray exposure.^44^ The membranes used in this study were 51 μm thick Nafion 112 (Dupont) cleaned with H_2_O_2_ and H_2_SO_4_, and the XPS beam power was set to 350 W. Understanding that there are distinct morphological, interfacial, and physicochemical characteristic differences between thick membranes (10–100s μm) and thin films (1–100s nm), differences in degradation under X-ray exposure should be expected as well.^13,14^ Paul et al. hypothesized damaging effects would be much more pronounced in a 10 nm Nafion thin film.^40^ Their study of thin films on model substrates succinctly observed substantial loss of F 1s and S 2p signal after 15 min of X-ray exposure with beam powers of 100, 200, and 300 W, indicating the degree of degradation is related to the X-ray beam itself. Consistent with sulfonic group loss, a corresponding suppression in protonic conductivity by as much as 90% was observed for the X-ray-exposed films.^40^ In the context of characterizing PEMFC CLs with XPS, Paul et al.’s work provides a more relevant comparison as the catalyst-ionomer interface measured by XPS will be at the information depth of standard lab-based XPS instruments, albeit with greater complexity due to the presence of catalyst and carbon support. Such studies of electrodes for PEMFCs are limited, but community interest in the analysis of PEM electrodes is growing, and therefore more definitive evidence illuminating when X-ray-induced damage to Nafion occurs and how to mitigate it is still needed.

This study investigates the impact of X-ray exposure during XPS measurements on the stability of a set of Nafion thin films and PEM electrodes by using three distinct XPS instruments. The sample set was designed and fabricated to include comparisons of Nafion films with different thicknesses and different local Nafion arrangements/structures. This was accomplished by varying the properties of the film’s substrate and including a Pt/C CL as a point of comparison. Following the initial study of the stability of Nafion-containing samples under different measurement conditions, this work presents a data acquisition approach that minimizes the impact of X-ray exposure-induced Nafion instability artifacts in XPS data. Application of this approach is demonstrated on both Nafion thin films and Nafion-containing CLs, reporting reliable data that can be used to investigate the differences (and lack thereof) in surface properties in Nafion-containing samples.

## 2 Methods

### 2.1 Materials

Nafion EW = 1000 was used in both thin films and CLs, obtained from Ion Power Inc. Nafion films were prepared by a well-established spin-casting method.^29,45^ Briefly, Nafion dispersion in isopropyl alcohol was diluted with additional isopropyl alcohol and sonicated until well-mixed, with different degrees of dilution used to control the film thickness. Spin coating was performed (5000 rpm and 30s) on the desired substrate, which in this work was either SiO_2_, glassy carbon (GC), or Pt.

CLs studied in this work featured a 46 wt % Pt catalyst supported on high-surface-area carbon (Pt/HSC, Tanaka TEC10E50E). CL fabrication was performed via previously established methods,^36^ in which a concentrated ink was prepared by dispersing catalyst and ionomer in a mixture of deionized water and isopropyl alcohol using a high-shear disperser (T25 Ultra Turrax, IKA). The ink was cast onto a gas diffusion medium, H23C8 (Freudenberg) via a Mayer Rod. CLs cast onto a gas diffusion medium were used instead of catalyst-coated membranes (CCMs) in order to avoid including the thick electrically insulating Nafion membrane in the sample to minimize confounding effects during XPS measurement.

### 2.2 XPS Measurements

#### 2.2.1 Instrumentation

The three XPS instruments employed in this study include a Kratos AXIS Supra (referred to as XPS-1), PHI *VersaProbe* III (referred to as XPS-2), and a custom Scienta-Omicron HiPP-3 environmental XPS system (referred to as XPS-3). XPS-1 is equipped with a monochromatic Al Kα operating at the same anode power (300 W) for both survey and high-resolution spectra. The survey spectra were acquired using a 160 eV pass energy, while the high-resolution core-level spectra were acquired using a 20 eV pass energy and the slot aperture open. For each spectrum, the data was averaged by specific cycles of scanning, as previously specified. XPS-2 is equipped with a scanning microprobe monochromatic Al Kα X-ray source. All spectra were acquired using a 100 μm X-ray source operating at 25 W of power. High-resolution spectra were obtained using a pass energy of 26 eV. A patented dual beam charge neutralization system utilizes both a cold cathode electron flood source (∼1 eV) and a very low energy ion source (≤10 eV) to provide charge neutralization. XPS measurements conducted with XPS-3 were performed with and without charge neutralization. The first set of experiments were conducted with both the X-ray beam on and the electron beam on for the duration of the measurements. An X-ray spot (oval) size of 900 μm was used, in this case resulting in a 300 W operating power. A low-energy electron flood gun was utilized to supply electrons to the sample surface to balance the positive charge generated by photoemission during a subset of measurements. Constant flood gun settings were applied, with an emission current of 50 μA and a voltage of 10.0 V. Samples were first outgassed in a UHV preparation chamber to a base pressure below 5 × 10^–9^ mbar. Pressure in the analysis chamber during measurement ranged between 2 × 10^–8^ and 4 × 10^–8^ mbar. The analyzer was maintained at or below a pressure of 1 × 10^–9^ mbar for all measurements and was operated in “Swift acceleration mode” which is described in detail elsewhere.^46^

#### 2.2.2 Stability Measurement Protocol

A common workflow for XPS measurement of Nafion stability with three unique XPS instruments (discussed above and referred to as XPS-1, XPS-2, and XPS-3) was established and used to acquire the data displayed in Figures 2–7. Following degassing and introduction to the analysis chamber, samples were first exposed to X-ray flux during sample focusing. In an effort to maintain uniformity, 5 min of X-ray exposure was allotted for focusing, and if less time was needed, the sample sat idle with X-ray exposure on until a total of 5 min from the initial exposure had elapsed. Then, a survey scan was performed (5.1 min). Following the survey, an initial scan of the F 1s (2.7 min) was collected to serve as a reference to confirm that no shifting in peak position was occurring at the time scale of the longer (13.1 min) S 2p scan. The first S 2p scan is considered and later referred to as the beginning of scan 1 and is followed by the F 1s and O 1s (4.4 min), and finally C 1s (4.8 min). This measurement sequence of S 2p, F 1s, O 1s, and C 1s was repeated 7 times, resulting in a total X-ray exposure time of 187.8 min (∼3 h and 8 min) for the total experimental duration. The cumulative X-ray exposure time for a given core level at the time of its completed data acquisition is shown in Table 1.

**Table 1:** XPS Stability Protocol Cumulative X-ray Exposure Time in Minutes

scan no.	S 2p	F 1s	O 1s	C 1s
1	25.9	28.6	33	37.8
2	50.9	53.6	58	62.8
3	75.9	78.6	83	87.8
4	100.9	103.6	108	112.8
5	125.9	128.6	133	137.8
6	150.9	153.6	158	162.8
7	175.9	178.6	183	187.8

Measurement settings were maintained from sample to sample, and as similar as possible between the three instruments. Samples were mounted on nonconductive double-sided tape (Kapton or similar) for experiments with the use of a charge neutralizer (CN). In the case of samples measured without charge neutralization, the samples were mounted on double-sided, conductive carbon tape. The insulating properties of the substrate were bypassed by taping over all 4 corners of the sample, providing a conductive pathway from the grounded sample holder to the sample surface. Survey scans were collected from 930–0 eV with a 1 eV step size. All high-resolution core levels were collected with a 0.1 eV step size. The F 1s spectrum spanned 10 eV and had one sweep. The S 2p and O 1s each were set at 12 eV wide, with 9 sweeps for the S 2p and 3 sweeps for the O 1s. The C 1s spectra were collected over a width of 15 eV and iterated with 3 sweeps. The dwell time for each step in both survey and high-resolution spectral measurements is 50 ms.

#### 2.2.3 Multispot Data Acquisition Protocol

An alternative data acquisition workflow aimed at minimizing spectral artifacts arising from X-ray damage was developed using multispot data analysis. It involved short scans at different areas of the sample, which were then summed to increase the signal-to-noise (S/N) ratio while minimizing X-ray exposure time for any one given area of the sample. The data acquisition protocol consisted of setting the F 1s to span 10 eV with 2 sweeps, the S 2p to span 12 eV with 8 sweeps, the O 1s to span 12 eV with 3 sweeps, and the C 1s to span over 15 eV with 2 sweeps. This protocol resulted in an ∼21 min X-ray exposure time for each area measured. The number of unique areas per sample measured varied depending on the S/N of the given sample, with a minimum of 5 areas acquired. The Nafion/SiO_2_ and/GC samples each only required 5 areas, while 8 and 9 areas were used for the Nafion/Pt and Pt/HSC samples respectively to ensure adequate S signal. Note that any sample focusing and survey spectra (not shown) were performed on separate areas than those used for any of the core-level measurements to ensure uniformity in X-ray exposure time between the areas on which core-levels were acquired.

#### 2.2.4 Data Processing

All data was processed using CasaXPS. For the sake of consistency, the first scan of the stability evaluation for all samples had their binding energy (BE) scale calibrated by setting the C 1s to 292.2 eV, based on the literature positions reported for Nafion membranes.^47^ All subsequent scans were then calibrated by the same amount as the initial scan in order to preserve shifts in the data that might arise due to either charging effects or a change in the chemical state. A Shirley background was used to estimate the region areas for all samples.

## 3 Results and Discussion

### 3.1 Nafion Composition: Considerations for XPS

**Figure 1:**
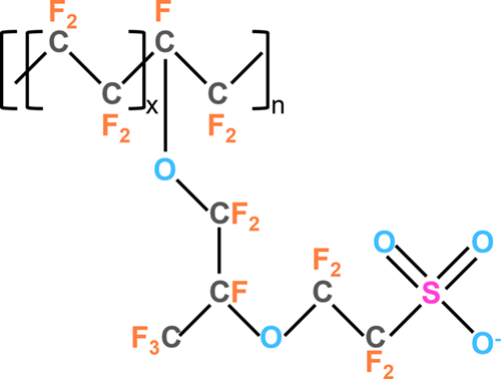
Schematic of a generalized Nafion molecular composition shown in its deprotonated form.

A generalized depiction of the Nafion molecule is displayed in Figure 1 to guide the discussion of possible spectral features arising from the various chemical states of S, O, F, and C present in Nafion. The S atom in the S 2p core level should occur in only a single chemical state since it is present only in the sulfonic acid group, which is responsible for proton conduction. However, the sulfonic acid group interacts with other sulfonic acid species in neighboring Nafion molecules to form ionic clusters, or with surface species of a catalyst (such as platinum) or support when present in a catalyst layer.^48,49^ While these interactions should not significantly change the sulfur’s bonding characteristics, it is certainly possible that the electron density surrounding the S atom will be perturbed by changes in that of the oxygen atoms during an interaction with another species, possibly resulting in small shifts in S 2p binding energy (BE). It should also be noted that the S 2p has a relatively small spin–orbital splitting that separates the 2p_3/2_ and the 2p_1/2_ by ∼1.2 eV, meaning that a single S species will appear as an asymmetric peak rather than 2 well-resolved features.

The next element to consider is oxygen, which is present only within the Nafion side chains as either a sulfonic acid species or as a linkage between a C–F and C–F_2_ species. The bonding of oxygen in this linkage is similar to that of an ether functional group and therefore will be referred to as “ether linkages” throughout this work. Additionally, each sulfonic acid species contains two oxygen atoms doubled bonded to sulfur and one oxygen present as a hydroxyl or an O^–^ single bonded to S depending on the environment. It is unclear if the ester-like oxygens in the sulfonic acid group will be clearly distinguishable from the hydroxyl species in the sulfonic acid group, however, it is expected that the ether linkages and the sulfonic acid oxygen species will be distinctly different. Indeed, among XPS studies of Nafion and Nafion-containing samples, there is a debate on the proper assignment of O 1s components to the two main oxygen species.^47^ We expect oxygen in the ether linkage to be shifted to a higher BE than that of oxygen bonded to sulfur due to the highly electron-withdrawing nature of the fluorine atoms bonded to the carbons in the ether linkage. Thus, we also expect the sulfonic acid signal to be present in a higher concentration than that of the ether linkage in the O 1s spectra for typical unmodified Nafion samples. It should be noted that a vast majority of common surface contaminants contain some oxygen species, which may cloud the interpretation of the O 1s spectra at times.

Fluorine, the most abundant element in Nafion, will primarily have a signal arising from the CF_2_–CF_2_ blocks comprising the Nafion backbone. Several other species are present in lower amounts, a CF_3_ species in the side chain, a CF species occurring alongside each ether linkage, and the HO_3_S–CF_2_ species at which the sulfonic acid group is attached to the side chain. Relative to the PTFE CF_2_–CF_2_ species, CF_3_ is likely to have the biggest difference in BE due to the increase in electron-withdrawing fluorine atoms without a change in other neighboring elements. However, due to the indistinct nature of electron sharing and the many competing factors in a large molecule like Nafion, it is difficult to predict how well-resolved these different C–F type species will appear in the F 1s.

Generally, the C 1s region is expected to have spectral features arising from similar differences in speciation to those of the F 1s. The strongest feature in the C 1s region will again be due to CF_2_–CF_2_ species primarily from the Nafion backbone. It is likely that less fluorinated species like the carbons attached to the ether linkage will be shifted slightly to lower BE. However, that C atom will still be bonded to two CF_2_ species and a F atom, likely making it very similar to carbon in the continuous CF_2_ chain. The CF_3_ species, which is also bonded to an ether linkage adjacent carbon, is perhaps more likely to be resolved from the CF_2_–CF_2_ due to its position as a terminal carbon, allowing the nature of the additional fluorine to have a greater influence on the carbon’s BE, and possibly shifting the peak to higher BE relative to CF_2_–CF_2_. It should be noted that no C–O bonds are present in isolation without the carbon bonded to oxygen and also containing bonds to fluorine, and therefore, the ether linkage should not be assigned to a BE representative of typical ether functional groups or other C–O species. This logic also must be applied to the sulfonic acid linking carbon atom, which is also bonded to 2 fluorine atoms and another CF_2_ species, meaning typical C–S BE values are not representative of the carbon bonded to sulfonic acid in Nafion. The C–O and C–S containing species are likely very close in BE to CF_2_–CF_2_, perhaps with a slight shift to lower BE due to the presence of fluorine bonding at the same carbon atoms. While C 1s is a region known for its presence of contaminants in the form of adventitious carbon and other species from ambient or processing history, such as solvent residue, the highly oxidized nature of C within Nafion should ensure that such contaminants are well resolved from the CF_*x*_ species characteristic of Nafion. Furthermore, many catalyst supports and some novel catalyst chemistries contain carbon, often in a graphitized or similar form. These species are also well resolved from CF_*x*_ species, enabling the study of electrode composition through investigation of the C 1s. It is evident that the Nafion molecule is rich with distinct chemical species and bonding environments. While not all will be able to be resolved from each other, several clear opportunities to correlate spectral features to changes in Nafion composition or orientation are possible, particularly in the O 1s and C 1s.

### 3.2 Effect of Instrumentation on Nafion Stability

**Figure 2:**
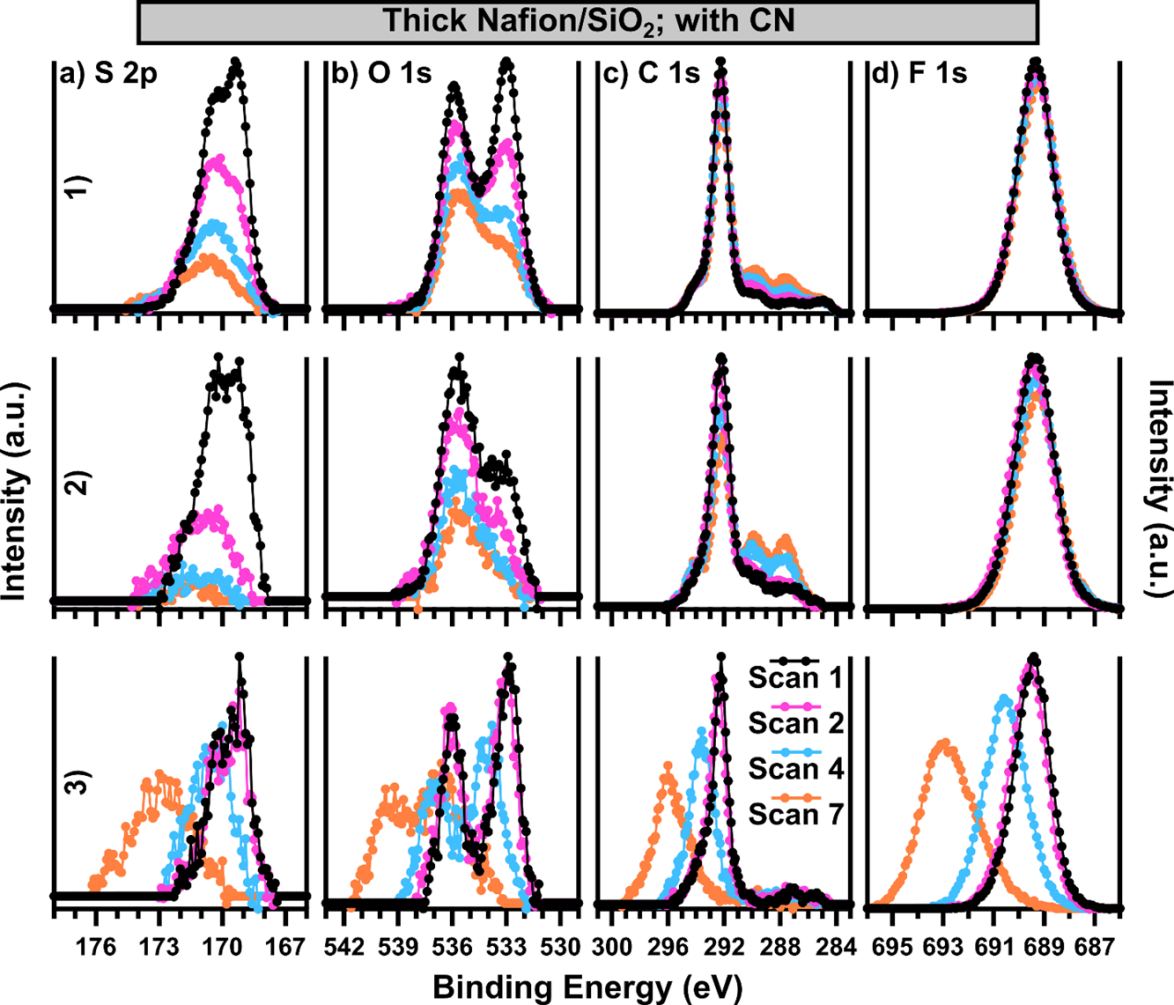
Each core-level (a) S 2p, (b) O 1s, (c) C 1s, and (d) F 1s of ∼120 nm thick Nafion films is displayed as a function of measurement iteration, with data from (1) XPS-1, (2) XPS-2, and (3) XPS-3 instruments featured. All data were collected with CN, and all spectra are background subtracted. Note that the BE range is extended relative to all other figures to encompass the shifts present in (3). BE calibration is not applied.

For measurement with three different XPS systems, Nafion films were cast onto SiO_2_ substrates, resulting in a set of thin (∼10 nm) and thick (∼120 nm) Nafion films—the literature suggests that ∼50 nm thickness is a critical point above which Nafion behavior changes.^13,50^ Impacts of both the X-ray beam and charge neutralization (CN) were evaluated by comparing measurements performed both with and without CN. The first experiment evaluated the changes in Nafion spectral features as a function of measurement time for ∼120 nm thick Nafion/SiO_2_ films, acquired with CN (Figure 2 and Table S1). While 7 iterative core-level measurement sequences were performed and measured to create the ∼ 3 h long experiment (Table 1), only the first, second, fourth, and seventh data points are displayed for visual clarity. Clear changes are detected as a function of cumulative X-ray exposure time for measurements with all three instruments, displaying major differences from instrument to instrument and core level to core level. The most significant changes are present in the S 2p core-level; Figure 2a1,a2 shows that the majority of S signal has disappeared by the end of the ∼3 h experiment, with Figure 2a2 displaying slightly more damage (95% decrease in S 2p area for 2-a2 vs 75% in 2-a1, Table S1). It is important to note that the majority of the loss of signal occurs between the first and second scans for both 2-a1 (32% decrease) and 2-a2 (57% decrease), while the data from the third instrument (2-a3) shows a much lower impact (10% decrease in S 2p area) from scan 1 to scan 2. However, in 2-a3, both the fourth and seventh scans show a decrease in overall signal and a shift to higher BE, most likely indicative of surface charge accumulation over time, despite the use of CN.

The O 1s spectra feature two main peaks, with the higher BE peak at ∼536 eV assigned to ether-linkages in the side chain and the lower BE peak at ∼533 eV attributed to the sulfonic acid group. The proportion of these peaks observed in the first scan is different for each instrument, with 2-b3 containing the highest initial amount of sulfonic acid and 2-b2 by far containing the least. While slight differences might be attributable to material heterogeneity and molecular orientation of the Nafion at the film’s surface, the extent to which 2-b2 has a significantly lower sulfonic acid signal in scan 1 compared to 2-b1 and 2-b3 suggests that significant sulfonic acid loss has already occurred during the first ∼25 min (Table 1) of the experiment. Considering the change over time, both 2-b1 (−55%) and 2-b2 (−70%) display an overall loss of the O 1s signal, with a disproportionate loss of the 533 eV peak. This indicates that Nafion damage occurs through a mechanism that results in at least partial but not complete loss of the side chain due to the decrease in both sulfonic acid and ether signal to different degrees. Figure 2b3 once again primarily indicates charging artifacts that increased over time, although damage may occur, as well.

Smaller changes are detected in the C 1s and F 1s spectra; however, some noteworthy phenomena still occur. In the C 1s, 2-c1 and 2-c2 show a decrease in the main CF_2_–CF_2_ peak, while an increase in two lower BE peaks at ∼289 and 287 eV occurs, resulting in relatively low net increases in C 1s area for most measurements (Table S1). These changes both indicate that side chain scission is occurring, resulting in a loss of some CF_*x*_ species, and the potential formation of new CF_*x*_ species (289 eV) or new C–O bonding modes depending on the nature of the degradation process. It is noteworthy that again, 2-c2 shows a slightly higher initial signal at ∼289 and 287 eV in the spectrum for scan 1. This further supports the idea that a significant amount of change is occurring within the 25 min between the conclusion of the first and second scans for this instrument. In contrast, 2-c3 primarily shows charging effects and does not have an increased low BE signal, further supporting that XPS-3 results in less damaged Nafion.

The amount of change in the F 1s region is small for 2-d1 and 2-d2, although some decrease in the signal is present by the end of the iterative measurement protocol. There is a slightly greater loss of signal in 2-d2 (−13%), consistent with the observations across all the elements in Nafion for this instrument. These observations are consistent with the hypothesis that damage is occurring to the side chain during XPS measurements, as both C and F show much less change over time due to their presence in the backbone as well. While there is a decrease in both the C and F signals for the third system, both 2-c3 and 2-d3 are again primarily impacted by shifts likely due to surface charge accumulation.

The results shown in Figure 2 confirm that Nafion films are damaged during the course of an XPS measurement, however, this damage occurs to a different extent and over a different time scale dependent on the instrument used. Since the X-ray power and spot size were not identical across all 3 instruments, an estimate for X-ray power normalized to the irradiation area on the sample is provided in Table 2. Calculating a more accurate value for X-ray flux/dosage is not straightforward as differences in emission current and focusing are likely present across the instruments. The first system (2-a–d1) resulted in significant damage, and the second system (2-a–d2) displayed even more damage as expected due to its ∼40× higher value in X-ray power per area. Some damage may be occurring from both XPS-1 and XPS-2 within the first 13–38 min of the experiment during acquisition of the data presented as Scan 1. The third system (2-a–d3) appeared to be mostly stable from the first to second scan, over a cumulative X-ray exposure time of 51 min despite being the intermediate case in terms of X-ray W/μm^2^. However, surface charge neutralization was not effectively maintained throughout the entire durability experiment for this system, resulting in both positive shifts and peak broadening in the later scans. There is a loss in signal for this instrument; however, this is convoluted due to the possibility that some of the signal loss is a result of ejected photoelectrons interacting with the localized electric field occurring at the surface due to charging which decreases the total signal. Therefore, it is difficult to definitively conclude whether actual damage to the Nafion film is occurring after measurement with XPS-3, or to what extent. Furthermore, there is a discrepancy between XPS-1 (lowest X-ray power per area) and XPS-3 (intermediate X-ray power per area) as XPS-1 causes more damage to the Nafion film. This suggests that additional factors such as CN hardware may make the relationship between X-ray power and Nafion damage complicated. The results in Figure 2 clearly demonstrate that change occurs within small enough time scales that high-resolution measurements of Nafion are effected by X-ray and CN exposure with damage and/or charging artifacts present if performed with standard acquisition times and protocols.

**Table 2:** Areal X-ray Power

XPS-1	XPS-2	XPS-3
1.2 × 10^–5^ W/μm^2^	4.0 × 10^–4^ W/μm^2^	5.0 × 10^–5^ W/μm^2^

**Figure 3:**
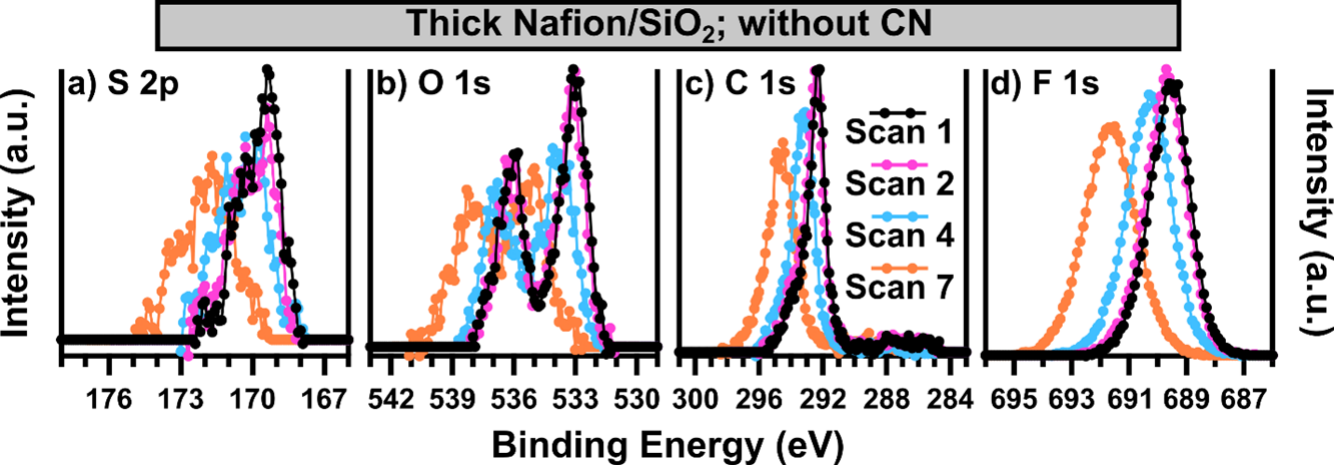
Each core-level (a) S 2p, (b) O 1s, (c) C 1s, and (d) F 1s of an ∼120 nm thick Nafion film is displayed as a function of measurement iteration, with data from XPS-3 featured. All data were collected without CN, and all spectra are background subtracted. BE calibration is not applied.

The next experiment investigated the influence of each system’s CN on the extent of damage to the Nafion films. The stability measurement protocol was applied to thick Nafion/SiO_2_ films in the absence of CN in each system. However, the surface charging for both the first and second instruments was prohibitively impactful, and stable data could not be obtained. The data for the third instrument are displayed in Figure 3, with the change in core-level area reported in Table S2. The results are very similar to those in Figure 2a–d3. Again, very little change occurs between the first and second scans, suggesting that with this instrument, a relatively stable window for acquisition does exist whether CN is used or not. However, measurement times exceeding ∼1 h begin to result in charging and/or damage to the Nafion film. Surprisingly, the shift in this case is slightly less without charge neutralization. It is possible that the difference in shift is due to film heterogeneity or that the dynamic nature of the Nafion film (chemical damage, nanoaggregate/nanodomain formation, or disruption) results in a change in the electronic environment of the Nafion over the course of the measurement.

**Figure 4:**
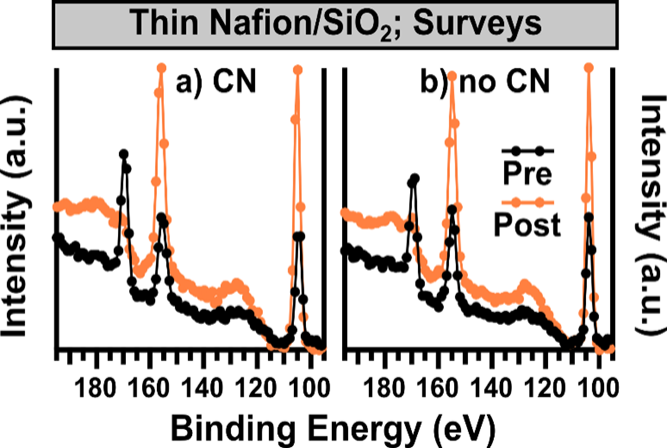
Subsection of the survey spectra collected using XPS-1 immediately prior to and immediately post stability protocol measurements are displayed for (a) measurement with CN and (b) without CN collected on thin Nafion/SiO_2_ films. No background correction or intensity normalization is applied.

The stability of thin Nafion films is investigated next. The thickness of these Nafion films (∼10 nm) is on the same order as the information depth limit of a typical XPS measurement, and therefore, it is possible that the SiO_2_ substrate may be detectable, particularly after the stability protocol measurement if film thinning is occurring. The surveys collected both prior to and after stability protocol measurements, with and without CN, using XPS-1 are shown in Figure 4. Data from 190 to 90 eV is shown so that the S 2p (∼169 eV), Si 2s (∼155 eV), and Si 2p (∼104 eV) are all displayed. Both Si peaks are present initially in a similar proportion to the S 2p peak, indicating that the Nafion films are likely at or below 10 nm in thickness. The change in proportion of the S and Si peaks poststability protocol measurement is striking, as in both cases the S 2p peak is hardly detectable relative to the background, and the Si peaks both display significant increases. This confirms that overall thinning of the Nafion thin film is occurring, and that the sulfonic acid species are particularly impacted. Additionally, it is important to note that the presence of O from the SiO_2_ substrate, both in the initial scan and as an increasing factor throughout the stability protocol measurement, will impact the interpretation of the O 1s results.

**Figure 5:**
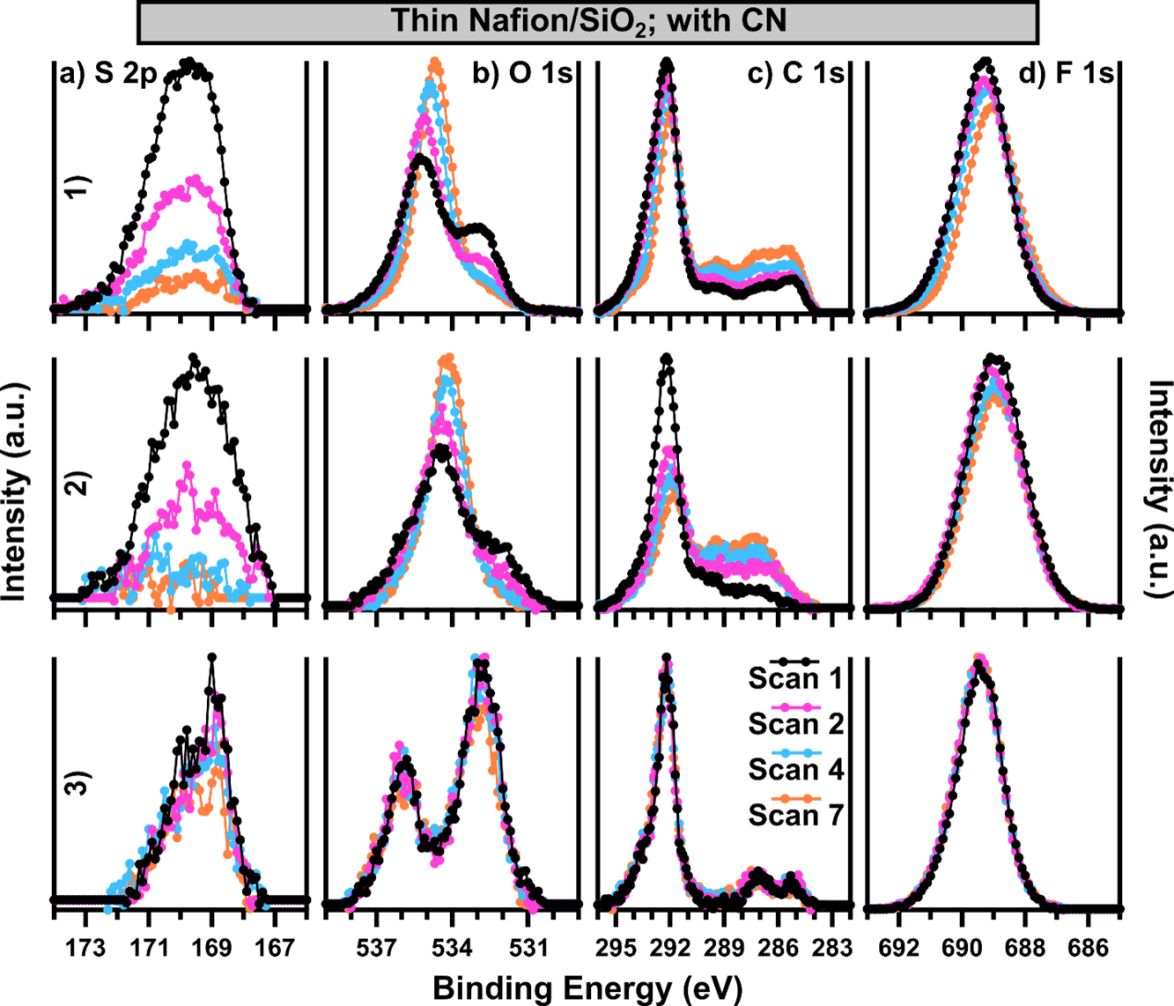
Each core-level (a) S 2p, (b) O 1s, (c) C 1s, and (d) F 1s of ∼10 nm thin Nafion/SiO_2_ films is displayed as a function of measurement iteration, with data from (1) XPS-1, (2) XPS-2, and (3) XPS-3 featured. All data were collected with CN, and all spectra are background subtracted. BE calibration is not applied.

The core-level spectra of thin Nafion films acquired with the use of CN by each instrument are displayed in Figure 5 (percent change in the core-level area is available in Table S3). Overall, the results for XPS-1 and XPS-2 are generally similar to those presented in Figure 2 for the thicker Nafion film, with a few key exceptions. Conversely, the results for XPS-3 are significantly different for the thin film compared to the thick film, as no charge accumulation artifacts are present in the thin Nafion film data. Looking first at the S 2p data, a clear difference in the peak shape is again present. The shape of the initial S 2p in 5-a3 has essentially the same asymmetric features as that of the S 2p in 2-a3, while 5-a1 and 5-a2 are both more rounded, symmetrical peaks. Similar to Figure 2, a much more dramatic loss in the S signal is present in both 5-a1 and 5-a2 (−81 and −93% over the entire experiment), while only a slight loss in the S signal occurs for 5-a3 (−9%). The dramatic loss of the S signal in 5-a1 is in good agreement with the survey results from Figure 4a.

Considering peak shape first, the O 1s, similar to the S 2p, has a significantly different shape in Figure 5b1,b2 compared to 5-b3, and the initial Scan 1 data are in Figure 2b1,b2. While the O 1s in Figure 5c3 is very similar to that in 2-b3 and 2-b1, as well as what is presented in literature examples,^47^ the O 1s in Figure 5b1,b2 deviates from the expected shape; specifically the lower BE sulfonic acid peak is lower intensity relative to that of the peak centered at ∼535 to 534.5 eV. This main peak is shifted ∼ 1 to 2 eV lower in BE than the ether peak in 5-b3, and all of the Scan 1 data in Figure 2b. Furthermore, this peak both shifts to lower BE and increases in intensity throughout the stability protocol measurements, while the lower BE sulfonic acid peak decreases in intensity, in agreement with the loss of the S signal in 5-a1 and 5-a2. This results in a near-zero percent change in the area of the O 1s for both 5-b1 and 5-b2, in stark contrast to the results for these instruments when thick Nafion films were studied. From the evidence of Si signal present both in the initial survey and the significant increase in the Si signal after the stability protocol measurements (Figure 4), along with the shifting position and increasing signal in Figure 5b1,b2, we attribute this feature to SiO_2_ signal from the buried interface of the Nafion/SiO_2_ substrate. As the Nafion film signal decreases due to possible film thinning, the signal from the SiO_2_ substrate increases; there is also a shift in its electronic environment due to the decrease in Nafion and perhaps a larger influence of CN on the substrate. This explanation also accounts for the different peak shape of the S 2p in Figure 5a1,a2, as it is likely that the S 2p is already being damaged in these cases, resulting in a change in S peak shape from an asymmetric to a more symmetric, rounded spectrum, in line with the features of the S 2p Scan 7 in 5-a3, where damage is occurring at a slower rate. Indeed, the O 1s in 5-b3 shows only slight damage over the duration of the experiment, resulting in a 9% decrease in the O 1s area.

Similar film thinning trends are observed for the C 1s and F 1s, without the complicating factor of contributions from the SiO_2_ substrate. XPS-1 and XPS-2 show similar results, with apparent decreases in the C and F signals, accompanied by slight progressive negative BE shifts. Additionally, both 5-c1 and 5-c2 show an increase in the lower BE C 1s features. The loss of F is more severe for XPS-2, with a 16% decrease in area compared to an 8% decrease in area for XPS-1. The change in area for the C 1s is not straightforward due to the increase of lower BE species, with both 5-c1 and 5-c2 resulting in near-zero percent change in area, although 5-c2 shows a more significant decrease in the CF_*x*_ feature. Identifying the chemical nature of the increasing lower BE peaks is challenging, as it is possible that they are present initially on the surface of the substrate as atmospheric contaminants or solvent residue and it is also possible that these represent solid degradation products of the Nafion film damage. It is interesting to note that these features extend to lower BE (likely more sp^3^ and sp^2^ carbon–carbon bonding) in 5-c1 while 5-c2 contains more signal in the region corresponding to carbon–oxygen and carbon–fluorine bonds. This may suggest slightly different degradation processes are occurring for the conditions resulting from these two different instruments; however, confidently identifying these species and relating them to degradation processes would require additional complementary investigations that are beyond the scope of this work. The nature of the negative BE shift observed for the F 1s and for the CF_*x*_ peak in the C 1s is also somewhat convoluted. It is likely that some or all of the origin of this shift can be attributed to the changing electronic environment of the sample, as photoemission and Nafion degradation occur under constant CN conditions, resulting in a dynamic electronic field at the surface of the sample during the stability protocol measurements.

Figure 5c3,d3 displays little to no change over the course of the stability protocol measurements, with very slight increases (likely within measurement error) in the C 1s and F 1s area observed. It is likely that with XPS-3 there is some slight damage to sulfonic acid groups but essentially no major loss of CF_*x*_ side-chain species of overall film thinning or damage to the PTFE backbone occurred. Overall, the results displayed in Figure 5 and Table S3 indicate that thin Nafion films are not stable in XPS-1 and XPS-2 by using standard acquisition conditions, with significant evidence that both side-chain damage and overall film thinning are occurring. There is significantly less change in core levels, especially the C 1s and F 1s, with XPS-3 compared to the other two instruments, but the loss in the S 2p area from the first scan (∼26 min) to the second scan (∼51 min) is still high at ∼17%. It is possible that this provides a large enough stable window for certain experiments/samples, however, it remains likely that the S 2p and O 1s regions would be impacted by typical measurements that occur over the course of hours.

**Figure 6:**
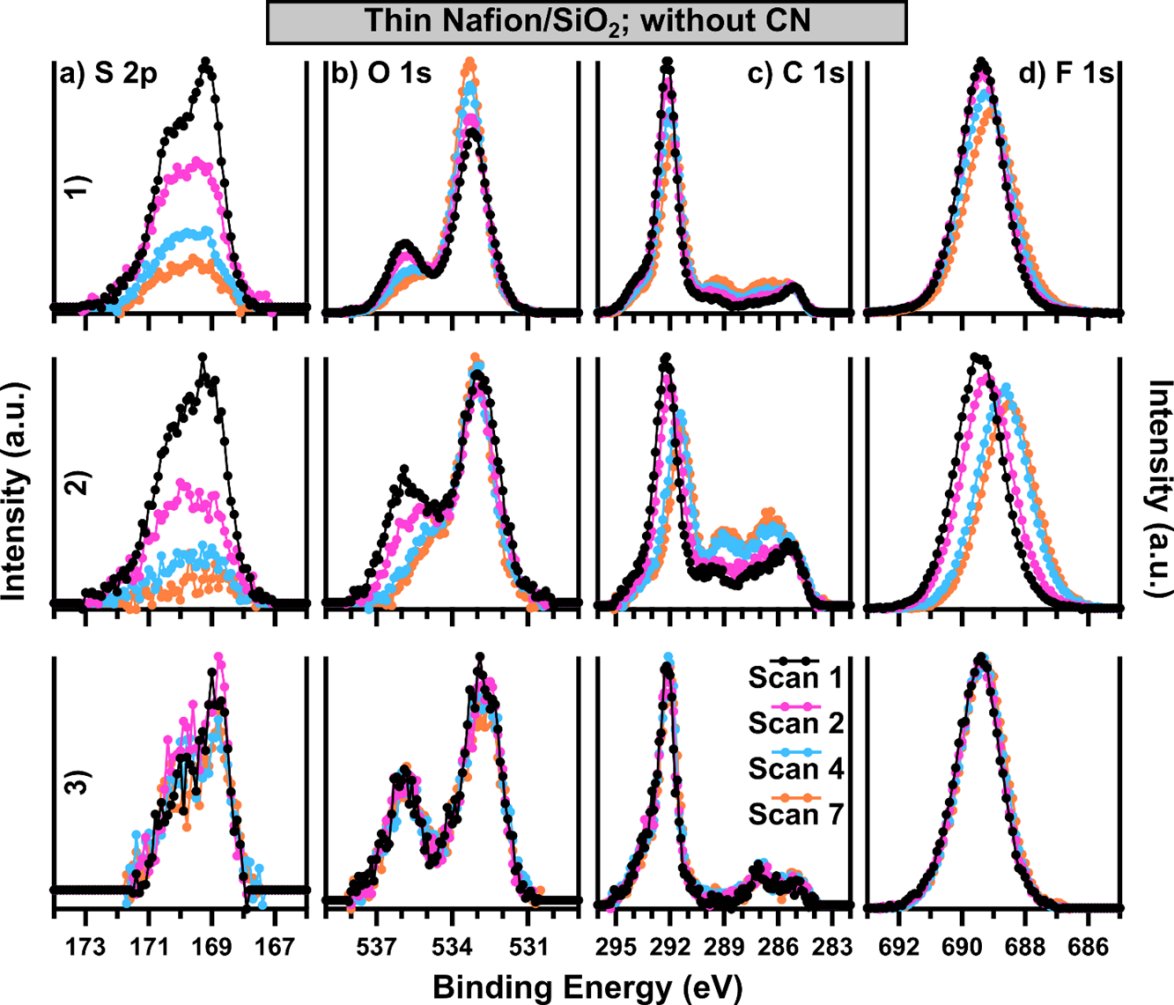
Each core-level (a) S 2p, (b) O 1s, (c) C 1s, and (d) F 1s of ∼10 nm thin Nafion/SiO_2_ films is displayed as a function of measurement iteration with data from (1) XPS-1, (2) XPS-2, and (3) XPS-3 instruments featured. All data were collected in the absence of CN, and all spectra are background subtracted. BE calibration is not applied.

In an effort to isolate the impact of CN on Nafion degradation during XPS measurements, stability protocol measurements were performed on a second set of thin Nafion/SiO_2_ films in the absence of CN (Figure 6). It is initially apparent that measuring thin Nafion films in the absence of CN results in less significant artifacts due to charge accumulation, as no significant positive BE shifts or peak broadening occurs, unlike the thick Nafion film measured in Figure 3. While the overall conclusion from Figure 5 that damage occurs more quickly in XPS-1 and XPS-2 than in XPS-3 still holds in the absence of CN, several key differences are noted. The first major difference is apparent in the features of S 2p in Scan 1 in both Figure 6a1,a2. In the absence of CN, the shape of the S 2p is more asymmetric, particularly in 6-a1. This is more in line with the S 2p collected by XPS-3 across the experimental conditions and more similar to the Scan 1 results on the thick Nafion films displayed in Figure 2a. This suggests that in the absence of CN, the onset of damage to the sulfonic acid group may be slightly later for XPS-2, and even more so for XPS-1. CN hardware generally features a focused electron beam, with some instruments also using a positive ion beam to aid in charge neutralization. XPS-1 features a focused electron beam located very near the sample; XPS-2 has a dual electron and Ar ion beam; XPS-3 has an unfocused low-energy electron beam. It is possible that the exposure to either of these stimuli significantly impacts the thin Nafion films, resulting in the difference in the initial S damage present in Figure 5a1,a2 versus 6-a1 and 6-a2. As the stability protocol measurement progresses, the S loss observed in 6-a1 and 6-a2 becomes very similar to that observed with CN, suggesting that the impact of the CN may be specific to initial exposure to the film. Interestingly, little to no difference between all core-levels for XPS-3 in Figures 5 and 6 is present. The CN in XPS-3 is a simple low-energy electron flood gun, with less ability to be focused on the sample and no ion beam. The lack of change between the use of the CN in this case versus XPS-1 and XPS-2, along with the differences in CN hardware suggest that the impact of the CN may be specific to the use of a positive ion beam, or due to the more focused nature of electron beam in these cases.

Both the initial features and the subsequent changes in features of the O 1s are different in the absence of CN as shown in Figure 6b1,b2 in contrast to Figure 5b1,b2. In the absence of the CN, the Scan 1 O 1s shows a much higher proportion (especially in 6-b1) of the lower BE feature that is indicative of sulfonic acid and may be overlapped by the contributions from the SiO_2_ substrate. Throughout the stability protocol experiment, the higher BE O 1s peak corresponding to ether linkages decreases, while the lower BE feature increases in 6-b1, and remains relatively constant in the case of 6-b2. This demonstrates the balance between the loss of the Nafion signal and the increase in the SiO_2_ substrate signal that is present as the Nafion film is damaged. The difference between the behavior of the Nafion/SiO_2_ thin films between the two instruments may indicate that sulfonic acid loss and overall film thinning may be occurring at different rates, as the loss of S is very similar between XPS-1 and XPS-2, while more O loss occurs for XPS-2 than XPS-1. It is also possible that slightly different thicknesses of Nafion films were measured, which would explain the relatively larger increase in lower BE 1s signal observed in 6-b1 if the region of Nafion film was slightly thinner, accentuating the impact of SiO_2_ substrate observed for this measurement. It is noteworthy that the position of the SiO_2_ in the absence of CN is much closer to the sulfonic acid species, with the SiO_2_ occurring just above 533 eV. In the case where the CN was used (Figure 5), the SiO_2_ feature was present at ∼534 to 534.5 eV and experienced a gradual shift to lower BE as the Nafion film was damaged. It is unsurprising that such a discrepancy exists, as the CN operates with static conditions, while the thickness and perhaps electronic properties, due to changing proportions of the side chain to the PTFE backbone and the presence of possible nanoaggregates with different conducting/insulating properties, of the Nafion thin film are changing throughout the experiment, subsequently changing the electronic environment of the SiO_2_ substrate as well. Overall, while the O 1s again presents a very complex, dynamic picture in 6-b1 and 6-b2, the results for thin Nafion films in the absence of CN are more in line with expectations and with the results on thicker Nafion films than the results presented with the presence of the CN. Also, in the case of XPS-3, little to no loss of the O 1s signal occurred, in good agreement with the results for this instrument in Figure 5, indicating that the CN in this instrument is not significantly impacting the stability of the Nafion films during measurement with XPS-3.

Some differences in the changes observed in the C 1s and F 1s in Figure 6 (without CN) compared with Figure 5 (with CN) do exist. While 6-c3 and 6-d3 are again very stable, XPS-1 and XPS-2 result in losses for both the C 1s and F 1s. The overall loss of CF_*x*_ species and F 1s species are very similar between the two instruments, which is apparent for F 1s in Table S4. In the case of the C 1s, the increase in lower BE C species makes a similar analysis difficult. Shifts to lower BE for the CF_*x*_ species and the F 1s species are present for both instruments, although the magnitude of the shift is greater in the case of XPS-2. Changes in the C 1s and F 1s will be caused by damage to the side-chain and the PTFE backbone, with the highest BE C 1s feature (294–293.5 eV) corresponding to CF_3_–O species present only in the backbone showing loss along with the CF_2_–CF_2_ species more indicative of backbone species. While it is impossible to isolate the loss of the side chain from the loss of the backbone due to the lack of resolution between CF_2_–CF_2_ species and other CF_*x*_ species besides CF_3_–O, it is likely that damage is occurring to both regions of the Nafion molecule. Ultimately, in the case of thin Nafion films measured in the absence of CN, it is apparent that a large stable window exists for measurement in the case of XPS-3, while XPS-1 and XPS-2 likely have some damage occurring in the first tens of minutes of the experiment.

In summary, the interlaboratory study of the stability of ∼10 nm thin and ∼120 nm thick Nafion films revealed several key findings. Consistent, with previous reports, Nafion films were found to experience damage during XPS measurements.^40,42−44^ Additionally, charging artifacts were detected, which depend on the properties of the Nafion film and the exact parameters of the XPS instrument used to conduct the measurement. While there are differences in the Nafion spectral dynamism over the course of the measurements apparently due to differences in Nafion film thickness and XPS instrumentation, it is very difficult to confidently designate differences in certain XPS components beyond a level of correlation and at a level of causation. It is possible that differences in X-ray flux due to different X-ray sources or X-ray focusing geometry and hardware are present, as it is also likely that different localized electronic environments at the surface of the sample are present due to differences in CN hardware. Based on findings with XPS-3 reported in Figures 5 and 6, experiments on the order of 1–3 h may result in damage/charging artifacts that are insignificant enough that adequate data could be acquired depending on the information desired and the resolution/data quality needed. However, it is clear from the comparison of the thick to thin Nafion films that the stable window of acquisition (if long enough to be useful) is likely to be sample-dependent. Therefore, it is clear from the results of this multi-instrument study that any such experiments, particularly if acquisition times beyond a few tens of minutes are used, must be preceded by an evaluation of the stability of a Nafion-containing sample under the XPS measurement conditions that are to be used in the planned experiment.

### 3.3 Investigation of Stability of Nafion-Containing Samples: Effects of the Substrate

With the promising results using XPS-3 in the absence of CN (Figure 6) suggesting that a stable acquisition window of up to 2 h exists before noticeable change occurs to the S 2p and O 1s of a Nafion/SiO_2_ thin film, we next investigated the stability of 3 additional Nafion samples under identical measurement conditions using the same instrument (XPS-3). This includes thin Nafion films cast onto glassy carbon (GC) and Pt substrates instead of the SiO_2_ substrates discussed in the previous section. Additionally, we included data for the CL made with Nafion and with a commercially available Pt/HSC which has been studied with XPS previously.^35,36^ The GC and Pt substrates serve as interesting comparisons to the SiO_2_ substrate, with Pt representing a surface that is more likely to attract sulfonic acid species, and the GC a more hydrophobic surface that is less likely to interact with sulfonic acid species than the SiO_2_ substrate. It is hypothesized that within such low-thickness domains (<10 nm) the influence of the interaction between Nafion and the substrate will be a significant driving factor of the Nafion film’s structure and morphology. While the presence of nanodomains and nanoaggregates in Nafion films dependent on the structure of the film has been posited in the literature,^10,13^ it is unknown if such features will be detectable with XPS, or if they will influence the stability of the thin film. Targeting substrates with opposite tendencies to attract the sulfonic acid headgroup of the Nafion side-chain may be a way to tease out differences in Nafion stability due to differences in film properties. Furthermore, including an actual Pt/HSC electrode will highlight any consideration toward XPS measurements when moving from studying model Nafion films to studying CLs with significantly different Nafion morphology.

**Figure 7:**
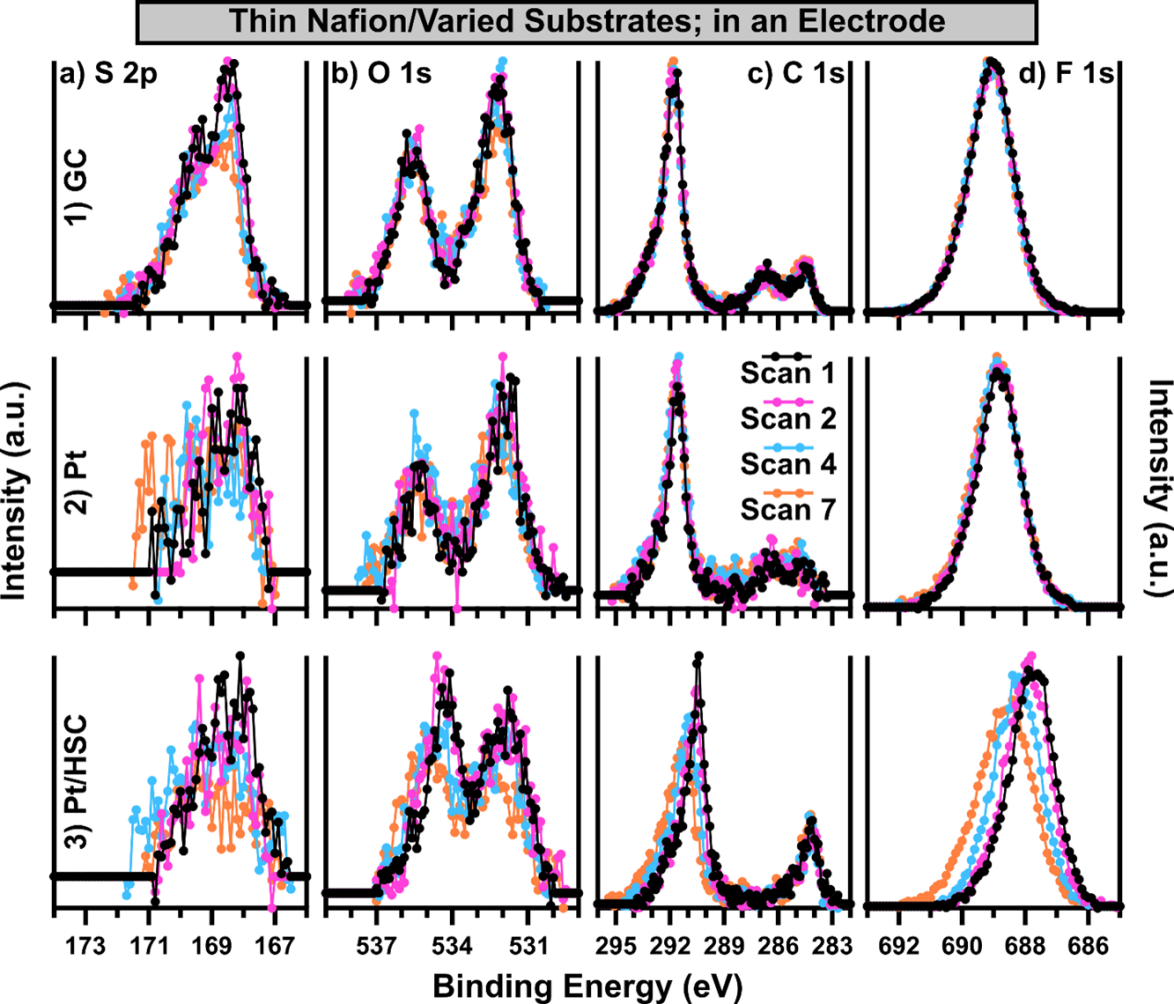
Each core-level (a) S 2p, (b) O 1s, (c) C 1s, and (d) F 1s of three Nafion-containing samples is displayed as a function of measurement iteration, with data from (1) a thin Nafion/GC film, (2) a thin Nafion/Pt film, and (3) a Pt/HSC electrode. All data were collected in the absence of CN using XPS-3, and all spectra are background subtracted. BE calibration is not applied.

The results of XPS stability measurements conducted on thin films (Nafion/GC, Nafion/Pt) and a Pt/HSC CL using the same stability protocol as before are presented in Figure 7 and Table S5. It is immediately clear that there is a difference in S/N for the S 2p among the samples. Scan 1 of the Nafion/Pt and Pt/HSC samples each appears to have a significantly lower sulfur signal than the Nafion/GC film, which appears more similar in both S/N and shape to the Nafion/SiO_2_ thin films reported in Figure 6. For the Pt/HSC electrode, this is easily attributed to the dispersed nature of the Nafion within a composite. Considering the Nafion/Pt thin film, lower S/N may be an indicator of stronger interactions between the sulfonic acid groups in the Nafion and the Pt substrate. However, it is also possible that this is the result of differences in film thickness if the Nafion/Pt area of analysis is thinner than that in the other samples. Indeed, comparing the S/N of the C 1s, which should not be as significantly impacted by the preferential molecular orientation of the Nafion sulfonic groups, there is also lower S/N for the CF_*x*_ species of the Nafion/Pt film compared to the Nafion/GC film. Therefore, in the context of Scan 1 of the stability protocol measurements, it is difficult to interpret whether any apparent differences between the Nafion/GC and Nafion/Pt films are reliable, emphasizing the need to develop a method to acquire high-quality data while avoiding Nafion damage. Generally, the two Nafion thin films display minimal impacts, with Nafion/GC displaying a near-zero change in the S 2p area from Scan 1 to Scan 2, and a 21% decrease in the S 2p area over the entirety of the experiment. The poor data quality of the S 2p from the Nafion/Pt thin film makes it difficult to estimate the extent of any sulfonic acid loss occurring; however, little change is apparent in the C 1s and F 1s, indicating that film thinning is not occurring.

The stability of the Nafion within the Pt/HSC electrode is worse than the stability of the thin ionomer films deposited on flat substrates. Some loss of signal is present at each core level, along with some positive shifting due to charging. Over the course of the stability protocol measurements, there is a 38% loss in the S 2p area and a 4% loss in the F 1s area. In the O 1s, contributions from surface oxidized species in both the Pt nanoparticles and the HSC support complicate the interpretation of the species, particularly those at lower BE where sulfonic acid species are overlapping with catalyst or support-bound oxygen. However, a loss in signal is still observed, along with a positive shift of the higher BE species, which likely is only representative of ether linkages in the Nafion side chain. Additionally, in the C 1s it is important to note that while CF_*x*_ species at higher BE display both loss of signal and positive shifts, the lower BE species representative of the carbon–carbon bonding from the HSC support display no change in position or signal. Such behavior suggests that domains may exist that experience differing localized electronic properties, causing some portion of the Nafion species to experience charge accumulation, while species associated with the carbon support are not impacted. Such a phenomenon may be dependent on the composition of the electrode (specifically, the proportion of Nafion to other species) and the properties of the catalyst and support. These results indicate that the stable acquisition window for ionomer-containing catalyst layers may be smaller than that of Nafion thin film samples and will likely vary depending on the properties of the electrode: nature of the catalyst, support, amount of ionomer, etc.

### 3.4 Demonstration of a Reliable Method for the XPS Measurement of Nafion

With the results of the previous sections demonstrating that acquisition of Nafion core-level data with XPS will likely contain significant adverse contributions due to damage during measurement in many situations, we now turn our focus toward demonstrating an XPS data acquisition method that relies on multispot analysis to minimize the effects of ionomer instability. Results presented in Sections 3.2 and 3.3 suggested a time window in which some data can be acquired before significant damage occurs; however, this time is likely too short to acquire data with sufficient S/N and resolution. Additionally, our findings demonstrated that the nature of the sample, instrument qualities, and acquisition parameters influence this time window. Short scans on multiple spots on the same sample are expected to avoid significant accumulation of error within the resulting spectra due to damage to the area of analysis during data acquisition. Assuming the sample is sufficiently homogeneous in terms of spatial variations of the surface composition, the resulting spectra acquired from multiple areas can then be summed to increase the S/N, essentially using a multipoint data acquisition similar to rastering or random spot modes available in other analytical instruments.

**Figure 8:**
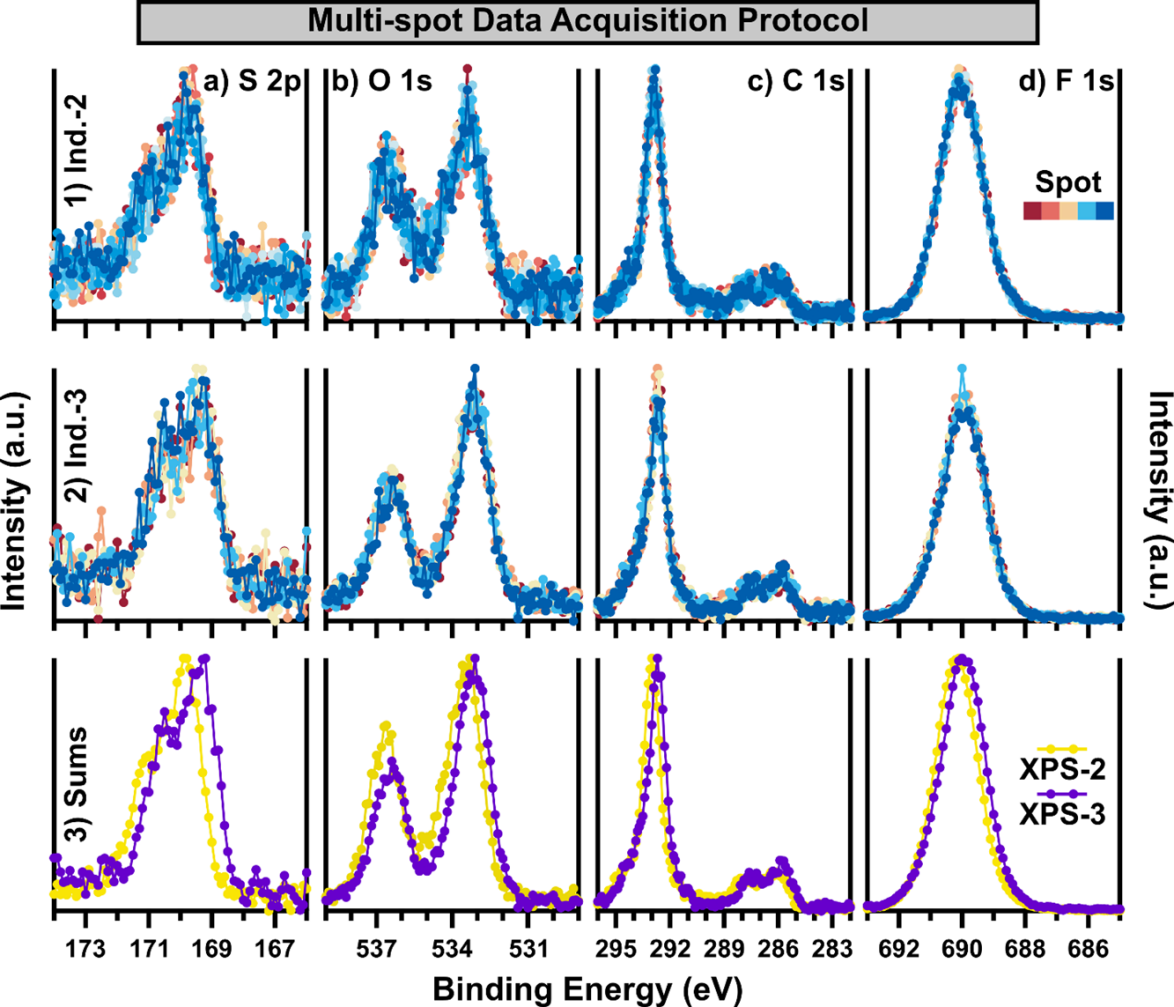
Each core-level (a) S 2p, (b) O 1s, (c) C 1s, and (d) F 1s of a thin Nafion/SiO_2_ samples collected using different data acquisition protocols is displayed. (1) Overlaid spectra collected from measurements of 10 unique areas on the sample with XPS-2 and (2) from 5 unique areas with XPS-3 are first displayed. In (1) and (2), no background correction, BE calibration, or intensity normalization is applied. Sums of the data for each instrument (3) are then displayed overlaid, with minimum–maximum intensity scaling applied to the data, while background correction and BE calibration are not applied.

Two instruments (XPS-2 and XPS-3) were used to acquire core levels of thin Nafion/SiO_2_ films from different areas on the same sample; the data obtained with each instrument is shown in Figure 8-1,-2 and compared by using overlays shown in Figure 8-3. In the case of Figure 8-1 featuring data from XPS-2, 10 areas were measured using short total acquisition times (10 min), whereas Figure 8-2 features 5 unique areas measured with slightly longer acquisition times (21 min per sequence) using XPS-3. The consistency of the features of the data is strikingly similar across the two instruments. While the S/N ratio of the individual S 2p and O 1s spectra is relatively poor in Figure 8-1a, -2a,-1b, and -2b, the summed data shown in Figure 8-3(a–d) display adequate S/N for each instrument. With X-ray exposure of each individual spot limited to under 10 and 21 min, respectively, this method of data acquisition avoids significant spectral artifacts due to Nafion degradation and charge accumulation, while also increasing the degree of spatial averaging of the overall measurement. In relatively spatially homogeneous samples, or those with randomly distributed spatial heterogeneity, increased area of spatial averaging can be considered a nonfactor or even an advantage in that it may result in a more representative measurement. Conversely, in samples with inhomogeneous compositions, for example at the edges of a coated composite sample which may vary from the composition of the rest of the sample, or samples with an intended spatial gradient, this method may be inappropriate or may require additional considerations. Additional insights related to S/N concentration can be gained from Figure S1 which displays an overlay of spectra resulting from the sum of 10 unique spots versus a sum of 100 unique spots with both sets of data collected on the same instruments (XPS-2). After normalizing the data, little to no difference in the S/N or spectral features is apparent. This may not be the case in samples with low S signal, such as CLs with low Nafion content. For such samples, a similar comparison can be conducted to determine the appropriate number of spots needed to produce a spectrum with sufficient S/N. Ultimately, Figure 8 demonstrates that it is possible to reliably collect XPS data on Nafion films while minimizing artifacts due to Nafion damage by minimizing X-ray exposure time through measurement of several unique areas on a sample and then summing the subsequent spectra.

### 3.5 Comparison of Physicochemical Properties of Nafion in Films and Electrodes

**Figure 9:**
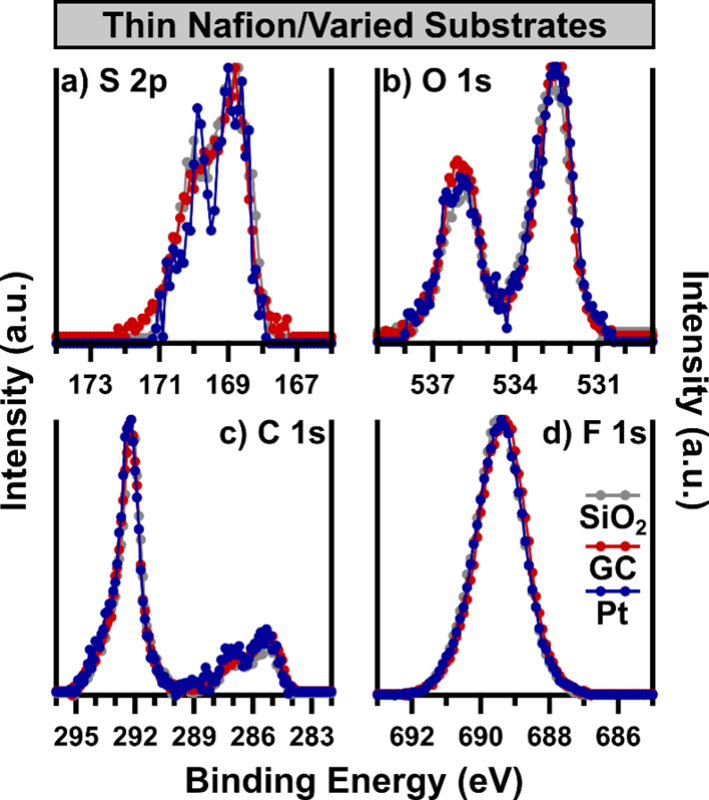
Each core-level (a) S 2p, (b) O 1s, (c) C 1s, and (d) F 1s of three Nafion-thin film samples collected using the multispot data acquisition protocol with XPS-3 is displayed. All data were collected in the absence of charge neutralization, and all spectra are background subtracted and minimum-maximum intensity scaled. All spectra are charge referenced by calibrating the C 1s to 292.2 eV.

With the necessary measurement approach development and validation completed, we now turn to the primary goal of conducting XPS characterization of Nafion: to identify similarities and differences in the surface physicochemical properties of several Nafion-containing samples. A first example is shown in Figure 9, where a comparison of three Nafion thin films cast on different substrates is made, comparing SiO_2_, GC, and Pt substrates. In the case of the three Nafion thin films with varied substrates, little to no difference can be discerned, indicating that the surface chemistry is not significantly impacted by the substrate. However, it can still be seen that the Nafion/Pt film has a lower S/N in the S 2p region than the other Nafion thin films.

**Figure 10:**
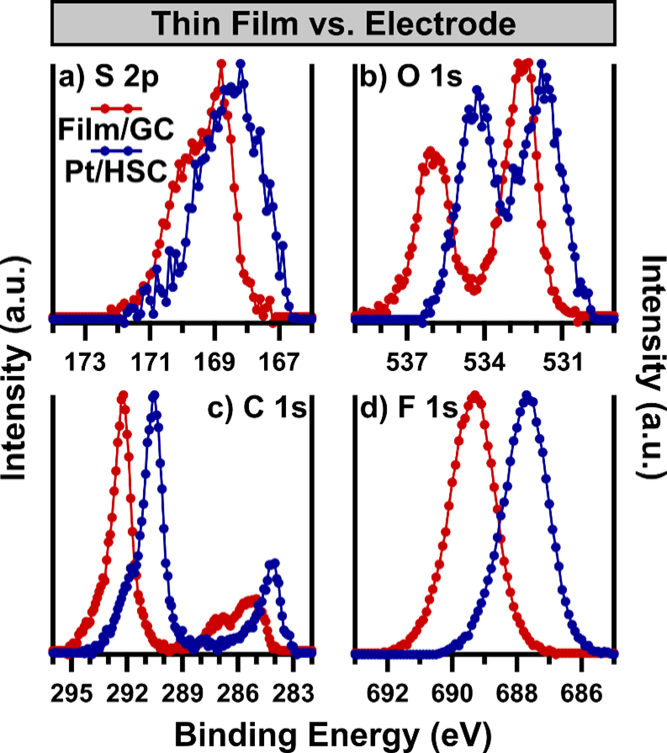
Each core-level (a) S 2p, (b) O 1s, (c) C 1s, and (d) F 1s of a Nafion-thin film on a GC substrate and a CL with a 50 wt % Pt/HSC catalyst are displayed. Data were collected using the multipoint data acquisition protocol with XPS-3 in the absence of charge neutralization. All spectra are background subtracted and minimum-maximum intensity scaled. The Nafion/GC thin film is charge referenced by calibrating the C 1s to 292.2 eV, while the Pt/HSC electrode is presented without charge referencing.

Next, we consider what effects incorporating Nafion into a CL may have on the Nafion surface properties. As the results shown in Figure 9 conveyed, little difference in the surface chemistry of Nafion thin films is present, and any of the 3 substrates would serve as a good basis for comparison to a Pt/HSC CL. Both the Pt and GC substrates represent model systems for the state-of-the-art Pt/HSC catalyst, however, better S/N is present in the Nafion/GC film, and so it is reproduced in Figure 10 for comparison. For consistency with previous figures and the literature, the Nafion/GC film is BE-corrected to set the C 1s peak maximum to 292.2 eV, a −0.3 eV shift from the as-measured data. No BE correction is applied to the Pt/HSC electrode due to the presence of a conductive Pt/HSC catalyst, as confirmed by a comparison of the C 1s position of the carbon support in Figure S2. It is immediately clear that significant shifts in peak maximum positions are present among all core levels, well beyond the BE charge correction applied to the Nafion/GC thin film. In all cases, the BE of the Pt/HSC electrode is shifted to a lower BE than that of the Nafion/GC film, however, differences in the magnitude of shift occur between the various core levels, which are shown in Table 3. Discerning the nature of these shifts is important to understand the changes in Nafion properties upon interacting with catalyst and support materials during incorporation into a CL. The starkly different environments of a continuous Nafion thin film and a Pt/HSC electrode in which Nafion is dispersed among and interacting with electronically conductive, metallic Pt and carbon may impact both the localized electronic environment and the chemical state of Nafion. The S 2p (Figure 10a) has a −0.6 eV relative shift in peak maximum and a slight change in the peak shape. The Nafion thin film has a clearly asymmetric character, whereas the Pt/HSC S 2p has a more rounded, symmetrical character with its peak maximum more centered within the spectrum, as opposed to that of the Nafion thin film, which has its peak maximum at its leading edge. As discussed earlier in this work, the nature of the spin–orbital splitting of the S 2p necessitates that a rounded, more symmetrical peak be indicative of multiple S chemical states. It is likely that the only interactions in the thin film sample are between neighboring sulfonic acid groups and other parts of the Nafion molecule interacting with a sulfonic acid group. In an electrode, the sulfonic acid species likely interacts with Pt at the surface of the catalyst and C at the surface of the support, in addition to the Nafion species. Since sulfonic acid-sulfonic acid interactions and sulfonic acid interactions with the PTFE backbone will be present in both samples, the difference in the S 2p peak shape of the two samples must be due to interaction with Pt catalyst or HSC support. Upon such interaction, an increase in signal shifted to slightly higher BE relative to that of the main S 2p_3/2_ peak would overlap the S 2p_1/2_, resulting in a more symmetrical spectrum with an asymmetric trailing tail at the highest BE end of the spectrum due to the second species S 2p_1/2_. This matches with the features of the Pt/HSC and contrasts with that of the Nafion thin film, allowing for an assignment of the lower BE species to sulfonic acid species that are experiencing Nafion–Nafion interactions, while the higher BE species is representative of sulfonic acid species in Nafion-catalyst or Nafion-support interaction. This assignment is convoluted with the overall shift in BE of all the core levels; however, the change in S 2p peak shape clearly delineates that a change in S chemical state or environment occurs upon incorporation of Nafion with Pt-based catalyst.

**Table 3:** Relative BE Shifts from the Pt/GC Thin Film to Pt/HSC CL

	S 2p	O 1s: ether	O 1s: SO_3_H	C 1s: CF_*x*_	F 1s
BE shift	–0.6 eV	–1.8 eV	–0.7 eV	–1.7 eV	–1.6 eV

Considering the O 1s next, a change in spectral features accompanies the overall shift in peak maximum positions. To evaluate the shift in the O 1s peak position, we first focus on the higher BE ether linkage peak as less change in peak shape occurs for this feature of the spectrum. The Pt/HSC electrode has shifted such that the higher BE peak has its maximum at ∼534.3 eV, shifted −1.8 eV relative to the Nafion/GC thin film. This shift is greater in magnitude than that of the lower BE feature; however, this is most likely due to overlapping signal from oxygen species arising from the surface of the Pt catalyst or the HSC support with that of the sulfonic acid; indeed, the lower BE feature in the Pt/HSC O 1s is asymmetric toward lower BE and broader than the corresponding feature in the Nafion/GC thin film. PtO_2_ or other surface Pt oxides will occur between 530 and 531.5 eV, in good agreement with the position of the asymmetric broadening observed at lower BE. The signal between 531 and 533 eV is challenging to assign due to the multiple possible surface oxide species when considering the carbon support, and due to the unknown severity of the negative shift of the sulfonic acid species in Pt/HSC relative to Nafion/GC. Based on the composition of Nafion, it would follow that the sulfonic acid group in the O 1s would have a similar shift as that of the S 2p, around −0.6 eV and significantly less than that of the O 1s ether-linkage. The shift is observed from a peak maximum at 532.5 eV in the Nafion/GC thin film to 531.7 eV in the Pt/HSC electrode, similar to the shift observed in S 2p. However, with multiple factors changing due to the inclusion of species from the catalyst and support, it is inadvisable to draw any definitive conclusions based on the magnitude of shift in the lower BE peak maximum position in the O 1s.

The main change in the C 1s when comparing Nafion in the Pt/HSC electrode to the Nafion/GC thin film is due to the presence of the HSC support, with an additional difference in a consistent shift in peak maximum position. A lower signal peak forms at low BE, in the case of an HSC support, where that peak is located at 284.2 eV. This position was verified by measuring the conductive Pt/HSC catalyst powder prior to incorporation into an electrode (Figure S2) while mounted on conducting tape to ensure no charge referencing was needed. The clear separation between the signal arising from the carbon support (284.2 eV) and the CF_*x*_ (290.5 eV) species present in Nafion allows for semiquantitative evaluation of electrode composition in terms of ionomer-to-carbon ratios, and studies featuring such analysis to track electrode composition have recently been published.^35,36^ There is a significant shift in the position of the CF_*x*_ peak maximum, from 292.2 eV for the Nafion/GC film to 290.5 eV for the Pt/HSC electrode. This −1.7 eV shift is very similar to that of the ether-linkage high BE peak in the O 1s.

The consistency in a shift of features ascribed to chemical species present in the Nafion backbone or side chain is further corroborated by the F 1s. While no significant change in peak shape is observed, the position of the F 1s in the Nafion/GC thin film is 689.3 eV, while the Pt/HSC F 1s is 687.7 eV (−1.6 eV shift) There is some uncertainty in the absolute values of these shifts, as a 0.3 eV increase in BE was applied to all core-levels of the Nafion/GC thin film for consistency in processing relative to the other Nafion films measured both with and without a CN in this study and for consistency with the literature. However, it is likely that measuring Nafion thin films, particularly on a conductive substrate such as GC, should be done without the presence of CN or BE adjustments, as the results of this comparison indicate that sample properties influence the position of characteristic Nafion peaks. In the case of a thin Nafion film vs Nafion dispersed with the catalyst that contains a conductive carbon support and metal Pt nanoparticles, significantly different Nafion interactions occur. In a thin film, Nafion can interact with only itself or the substrate and only a small portion of the film can interact with the substrate. The sulfonic acid group may interact with other Nafion molecules or, perhaps more likely, will interact with other sulfonic acid groups in neighboring Nafion molecules. Distinct nanoaggregates can form, with higher concentrations of sulfonic acid in some regions and higher concentrations of PTFE backbones in others. This does not represent a particularly electronically conductive environment, likely resulting in charging during XPS measurements. However, perhaps due to the small size scale of the film thickness, the proximity to an electronically conductive substrate or conductive tape, or the properties of Nafion itself, this electronic charging phenomenon arises in a uniform nature, resulting in equilibrium peak positions generally like those of bulk Nafion membranes. In a CL, the dispersed nature of the Nafion molecules with the catalyst and pores inherently results in a more heterogeneous surface, likely influencing the surface electric field resulting from photoemission during measurement. The inclusion of electronically conductive Pt and HSC also creates a much more electronically conductive bulk material while changing the nature of the molecular and nanoscale interaction of Nafion. The result is a significant shift to lower BE (indicative of less charging or negative charge transfer due to interaction with a conductor), most dramatic for that of PTFE backbone species which, especially when aggregated, are poor electronic conductors. Further experimentation is needed to thoroughly support this possible explanation; however, the evidence clearly points toward differences in the electronic environment resulting in detectable shifts in Nafion XP spectra, and it is likely that these differences arise from the character of Nafion interactions within a sample. These two examples, one comparing the effect of substrates on interactions with thin Nafion films and the second comparing interactions in a thin film with those observed in a CL, clearly indicate the potential of XPS for the identification of physicochemical differences in Nafion-containing samples.

## 4 Conclusions

This multi-instrument study first investigated the stability of Nafion-containing samples as a function of XPS measurement conditions, with the results indicating that Nafion films degrade under typical XPS measurement conditions, resulting in loss of the proton-conducting sulfonic acid species, possibly due to partial scission of the side chain. Measurement with charge neutralization employed exacerbated this damage, damaging the film considerably during the time that it took to focus on a sample and conduct the first core-level measurements. These results demonstrate that Nafion degradation occurs on the order of tens of minutes of measurement time, causing artifacts in the data that prevent a reliable interpretation of the results. This study shows that the exact time at which spectra of Nafion-containing samples will no longer be reliable will depend on both the characteristics of the XPS instrument and the acquisition parameters used. Additionally, the properties of the sample itself influence the stability. For example, Nafion in an electrode was shown to degrade faster than samples containing a thin Nafion film. XPS measurements of Nafion-containing samples must be performed by first evaluating their stability to definitively ensure that Nafion degradation artifacts in their data are minimized and data are interpreted correctly. Therefore, a simple and robust data acquisition method involving short scans on multiple unique, fresh areas of a sample and a summary of the resulting data was demonstrated. This protocol was shown to provide reliable XPS measurement of Nafion-containing samples and can increase the spatial averaging of the measurement, resulting in a more representative data point than a scan of a single area. However, this method must be used with caution when dealing with heterogeneous samples.

Finally, with a method for reliable data acquisition established, case studies comparing Nafion films supported on three different substrates and evaluating Pt/HSC electrodes were performed to show differences in the physicochemical properties of Nafion in different samples. Significant shifts in the Nafion spectral features between the composite electrode and thin Nafion film were attributed to a change in the local electronic environment of Nafion when it interacts with a conductive catalyst or support material. Ultimately, this work serves as a guide for reliable XPS measurement of Nafion to enable a better understanding of the role that Nafion plays in various applications. These results motivate further studies using XPS to compare Nafion-containing samples as a function of material processing, testing conditions, and in various in situ environments for applications in PEM fuel cells and electrolyzers.

## Supporting Information Available

Tables of peak area loss corresponding to stability figures, comparison of multispot acquisition with a different number of unique areas, and comparison of the C 1s from a Pt/HSC catalyst powder and electrode (PDF)
