# Supplementary material for: X-ray Photoelectron Spectroscopy Analysis of Nafion-Containing Samples: Pitfalls, Protocols, and Perceptions of Physicochemical Properties
Source: J Phys Chem C Nanomater Interfaces. 2024 May 10;128(20):8467–82. doi: 10.1021/acs.jpcc.4c00872 (PMC11129293; doi:10.1021/acs.jpcc.4c00872)
Supplement: Supplementary file 1 — jp4c00872_si_001.pdf [file jp4c00872_si_001.pdf]

X-ray Photoelectron Spectroscopy Analysis of Nafion-Containing Samples: Pitfalls, Protocols, and Perceptions of Physicochemical Properties

Authors: Michael J. Dzara,[1†] Kateryna Artyushkova,[2] Jayson Foster,[1] Hamideh Eskandari,[3] Yechuan Chen,[4] Scott A. Mauger,[5] Plamen Atanassov,[4] Kunal Karan,[3] and Svitlana Pylypenko[1,5*]

[1]Colorado School of Mines, Department of Chemistry, Golden CO, 80401, United States

[2]Physical Electronics Inc., East Chanhassen MN, 55317, United States

[3]University of Calgary, Department of Chemical and Petroleum Engineering, Calgary AB, T2N 1N4, Canada

[4]University of California Irvine, Department of Chemical & Biomolecular Engineering, Irvine CA, 92697, United States

[5]National Renewable Energy Laboratory, Materials Science Center, Golden CO, 80401, United States

[†]Present Address: National Renewable Energy Laboratory, Chemical and Materials Science Center, Golden, CO, 80401, United States

[*]Corresponding author, spylypen@mines.edu or Svitlana.pylypenko@nrel.gov

Tables of percent change (a decrease is negative) in the area of each core-level relative to the area of that core-level measured in the first scan are displayed for each Nafion stability-protocol measurement set reported.

Table S1: Thick Nafion/SiO_2; with CN

	XPS-1				XPS-2				XPS-3			
	S	O	C	F	S	O	C	F	S	O	C	F
2	-32%	-18%	5%	1%	-57%	-27%	-1%	-1%	-10%	3%	1%	0%
4	-60%	-42%	9%	1%	-88%	-55%	5%	-6%	-17%	-14%	3%	-1%
7	-75%	-55%	12%	-2%	-95%	-70%	4%	-13%	-13%	-10%	-5%	1%

Table S2: Thick Nafion/SiO_2; without CN

	XPS-3			
	S	O	C	F
2	-9%	-3%	6%	4%
4	-3%	-10%	-2%	5%
7	-11%	-7%	4%	6%

Table S3: Thin Nafion/SiO_2; with CN

	XPS-1				XPS-2				XPS-3			
Scan #	S	O	C	F	S	O	C	F	S	O	C	F
2	-27%	1%	2%	1%	-56%	-5%	0%	-2%	-17%	-2%	4%	3%
4	-65%	1%	-1%	-2%	-84%	-7%	0%	-11%	-13%	-2%	7%	3%
7	-81%	4%	-4%	-8%	-93%	-1%	3%	-16%	-33%	-9%	5%	5%

Table S4: Thin Nafion/SiO_2; without CN

	XPS-1				XPS-2				XPS-3			
Scan #	S	O	C	F	S	O	C	F	S	O	C	F
2	-46%	-3%	2%	-3%	-45%	-17%	2%	-2%	14%	-3%	4%	-1%
4	-74%	-3%	2%	-9%	-77%	-29%	8%	-7%	1%	-4%	10%	0%
7	-88%	-2%	2%	-17%	-90%	-34%	8%	-12%	-7%	-7%	4%	3%

Table S5: Thin Nafion/Varied Substrates; in an Electrode

	/GC				/Pt				Pt/HSC			
Scan #	S	O	C	F	S	O	C	F	S	O	C	F
2	0%	6%	0%	-3%	2%	9%	15%	2%	-18%	7%	-6%	1%
4	-8%	3%	-3%	-3%	-12%	15%	32%	-12%	1%	-9%	-2%	-1%
7	-21%	-3%	2%	-3%	17%	3%	23%	17%	-38%	-17%	1%	4%

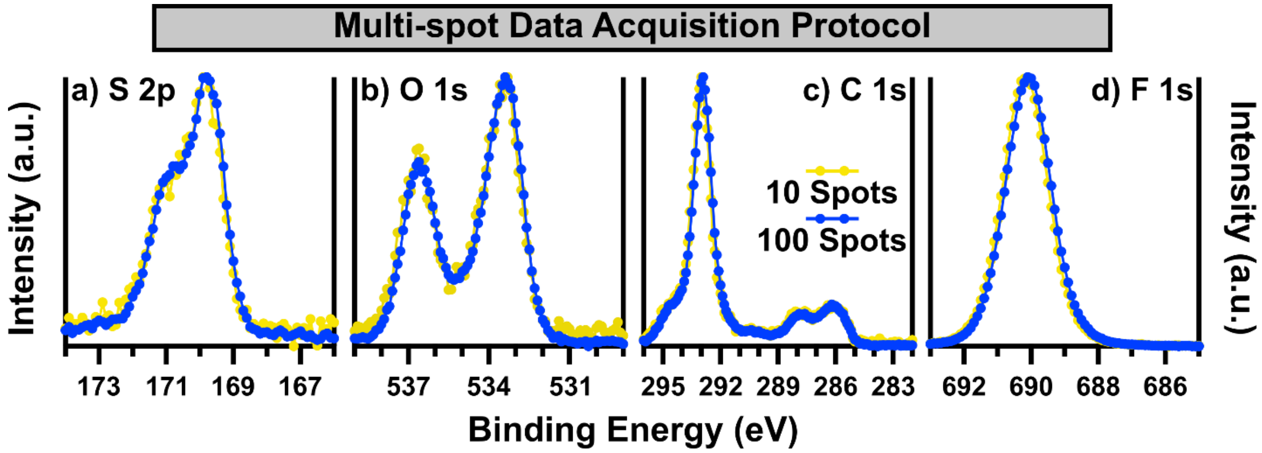

Figure S1: Sums of each core-level (a) S 2p, (b) O 1s, (c) C 1s, and (d) F 1s of a thin Nafion/SiO_2 samples collected using a different number of unique areas of analysis with XPS-2 are displayed. Minimum-maximum intensity scaling is applied to the data, while background correction and BE calibration is not applied.

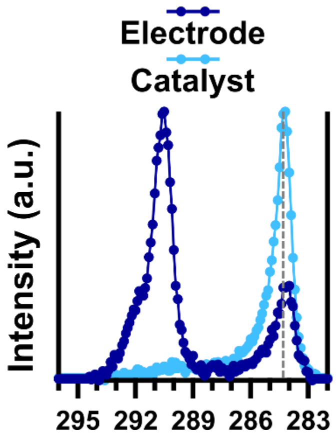

Figure S2: The C1s is displayed for a Pt/HSC catalyst powder alongside the same catalyst integrated with Nafion in an electrode. The position of the HSC is highlighted with a dashed line to show no shift in BE occurs for the HSC between the two samples.
